# Polycystic Ovary Syndrome in Insulin-Resistant Adolescents with Obesity: The Role of Nutrition Therapy and Food Supplements as a Strategy to Protect Fertility

**DOI:** 10.3390/nu13061848

**Published:** 2021-05-28

**Authors:** Valeria Calcaterra, Elvira Verduci, Hellas Cena, Vittoria Carlotta Magenes, Carolina Federica Todisco, Elisavietta Tenuta, Cristina Gregorio, Rachele De Giuseppe, Alessandra Bosetti, Elisabetta Di Profio, Gianvincenzo Zuccotti

**Affiliations:** 1 Pediatric and Adolescent Unit, Department of Internal Medicine, University of Pavia, 27100 Pavia, Italy; elisavietta.tenuta01@universitadipavia.it; 2 Pediatric Department, “Vittore Buzzi” Children’s Hospital, 20154 Milan, Italy; elvira.verduci@unimi.it (E.V.); vittoria.magenes@unimi.it (V.C.M.); carolina.todisco@unimi.it (C.F.T.); alessandra.bosetti@asst-fbf-sacco.it (A.B.); elisabetta.diprofio@unimi.it (E.D.P.); gianvincenzo.zuccotti@unimi.it (G.Z.); 3 Department of Health Sciences, University of Milano, 20142 Milano, Italy; 4 Laboratory of Dietetics and Clinical Nutrition, Department of Public Health, Experimental and Forensic Medicine, University of Pavia, 27100 Pavia, Italy; hellas.cena@unipv.it (H.C.); cristina.gregorio@unimi.it (C.G.); rachele.degiuseppe@unipv.it (R.D.G.); 5 Clinical Nutrition and Dietetics Service, Unit of Internal Medicine and Endocrinology, ICS Maugeri IRCCS, 27100 Pavia, Italy; 6 Department of Biomedical and Clinical Science “L. Sacco”, University of Milan, 20157 Milan, Italy

**Keywords:** polycystic ovary syndrome, fertility, obesity, adolescents, nutrition, diet, food supplements

## Abstract

Polycystic ovary syndrome (PCOS) is the most common endocrine disorder in young reproductive-aged women. PCOS is often associated with obesity and impairs reproductive health. Even though several theories have been proposed to explain the pathogenic mechanism of PCOS, the role of insulin resistance (IR) as a key etiological component, independently of (but amplified by) obesity, is well recognized. The consequent hyperinsulinemia activates excessive ovarian androgen production, leading to PCOS. Additionally, the state of chronic inflammation related to obesity impacts ovarian physiology due to insulin sensitivity impairment. The first-line treatment for adolescents with obesity and PCOS includes lifestyle changes; personalized dietary interventions; and, when needed, weight loss. Medical nutrition therapy (MNT) and the use of specific food supplements in these patients aim at improving symptoms and signs, including insulin resistance and metabolic and reproductive functions. The purpose of this narrative review is to present and discuss PCOS in adolescents with obesity, its relationship with IR and the role of MNT and food supplements in treatment. Appropriate early dietary intervention for the management of adolescents with obesity and PCOS should be considered as the recommended approach to restore ovulation and to protect fertility.

## 1. Introduction

Polycystic ovary syndrome (PCOS) is the most common endocrine disorder in young reproductive-aged women. PCOS is a multifactorial disorder and is characterized by a combination of clinical (anovulation and hyperandrogenism), biochemical (excessive androgen and luteinizing hormone concentrations) and ovarian morphological (polycystic ovaries) features [1,2]. PCOS is often associated with obesity and impairs reproductive health. 

Even though several theories have been proposed to explain the pathogenic mechanism of PCOS, the role of insulin resistance (IR), independently of (but amplified by) obesity, is a key etiological component. IR is associated with consequent hyperinsulinemia, which activates excessive ovarian androgen production, leading to PCOS development. 

Additionally, obesity-related inflammation may have potential implications for ovarian physiology due to the dysregulated adipokine secretion, affecting insulin sensitivity [2].

In adolescents with obesity, increased visceral adiposity is also associated with hormonal changes impairing the hypothalamus and the pituitary function and directly affecting ovarian function. 

PCOS in adolescence still remains a great clinical issue from the diagnostic and therapeutic points of view. However, early diagnosis and treatment of PCOS are crucial to restore ovulation [3]. The first-line treatment for adolescents with PCOS is lifestyle changes, including nutrition treatment, physical activity and behavioral therapy. Medical nutrition therapy (MNT) in these patients aims at improving symptoms and signs, including insulin resistance and metabolic and reproductive functions, by means of personalized dietetic treatment considering energy restriction as well as the macro- and micronutrient composition of the diet, which, regardless of weight loss, impacts insulin sensitivity. 

IR is considered a metabolic precursor of PCOS development [1,2]. The purpose of this review is to present and discuss PCOS in adolescents with obesity, emphasizing its relationship with IR and the role of MNT and food supplements in treatment. Early detection of and nutritional intervention in modifiable risk factors, such as IR, should be considered as the recommended approach to restore ovulation and to protect fertility in adolescents with obesity and PCOS. Considering the direct interaction between nutritional elements and other risk factors, such as oxidative stress and gut microbes, the benefits of nutrition therapy and food supplements may also be crucial in the absence of IR.

## 2. Methods

This narrative review aims to address PCOS treatment in adolescents with obesity, its relationship with IR and the role of MNT and functional foods. The authors V.C.M., C.F.T., E.T., C.G., R.D.G. and E.D.P. independently identified the most relevant original scientific papers, clinical trials, meta-analyses and reviews published in the past 15 years, in the English language. The electronic databases PubMed, Scopus, EMBASE and Web of Science were used for this research. The following keywords were used: polycystic ovary syndrome, insulin resistance, hyperinsulinism, hyperandrogenism, obesity, diet, nutrition, obesity or diet in polycystic ovary syndrome, lifestyle intervention, menstrual irregularities, nutraceuticals or inositol or docosahexaenoic acid and polycystic ovary syndrome, functional foods or food supplements or bioactive compounds and insulin resistance. The contributions were critically reviewed by V.C., E.V. and H.C. and collected by V.C., E.V., H.C., V.C.M., C.F.T., E.T., C.G., R.D.G., A.B. and E.D.P. The resulting draft was discussed with all co-authors [4]. The final version was then recirculated and approved by all.

As a narrative review, several statements based on expert opinions and not evidence-based or supported by appropriate clinical trials are included.

## 3. Polycystic Ovary Syndrome in Adolescents

PCOS is a complex and heterogeneous disorder that affects women of reproductive age and often starts to manifest during adolescence. Although PCOS is a common condition, its etiology and progression remain unclear [5].

In the ovarian physiology, a dynamic balance between growing and dormant follicles exists; the factors influencing follicular growth are coordinated, and only a single follicle is selected for terminal maturation and ovulation. In adolescents with PCOS, an imbalance between androgens, anti-Müllerian hormone (AMH) and follicle-stimulating hormone (FSH) has been reported; the process of follicular development is characterized by relative luteinizing hormone (LH) increase, promoting androgen production [5,6] and inadequate FSH secretion, leading to a decreased conversion of androgen to estradiol and follicular growth arrest [7].

Adolescents with PCOS often have hyperinsulinemia and insulin resistance (IR). Interestingly, these factors have been demonstrated to be correlated to hyperandrogenism, as increased androgen levels promote IR and hyperinsulinemia, which, in turn, promote androgen [8] and LH [5] secretion, respectively, by adipose tissue and the pituitary gland [5].

Thus, among the factors involved in the pathogenesis of this condition, the most characteristic seem to be abnormal ovarian steroidogenesis and folliculogenesis [8,9], IR and hyperinsulinemia [9,10,11] and reproductive neuroendocrine dysfunction [11,12]. The exact pathophysiological roles of these factors have not been elucidated yet, but recently [11], the pubertal ontogeny of PCOS has been addressed considering the abnormal development in girls at risk—that is, daughters of women with PCOS (PCOSd), girls with premature pubarche (PP) and those with obesity.

Specifically, daughters of women with PCOS were found to have higher AMH, insulin and androgen concentrations compared to controls [13,14,15]. Moreover, PCOS cases showed higher ovarian volume [13] and increased estradiol and LH responses to gonadotropin-releasing hormone (GnRH) stimulation [16], typical metabolic traits of PCOS [17,18,19].

Girls with PP, a hallmark of adrenarche, showed PCOS-like disturbances such as oligomenorrhea, hirsutism and biochemical and functional hyperandrogenism in response to acute GnRH stimulation across puberty and in the postpubertal period [20]. Moreover, girls with PP also presented increased levels of AMH [21,22,23].

Hyperinsulinism was hypothesized to link PCOS to PP, triggering androgen hypersecretion by the ovaries and adrenal glands [24,25].

Lastly, girls with obesity exhibited higher testosterone levels compared to controls [15], and weight loss was found to improve PCOS clinical features [26]. Moreover, childhood obesity has been correlated with increased PCOS risk as a genetic function [27]. Even in this case, one important culprit seems to be IR [28].

Interestingly, small-for-gestational age (SGA) babies (defined as neonates born with a weight lower that two standard deviations below the mean or <10th percentile for the gestational age) are considered to be at risk of hyperandrogenism, IR, PP and PCOS. Therefore, pubertal development monitoring is recommended [29].

The mechanisms behind this association remain poorly known, and it is agreed that further studies should address the common origin among these conditions (during fetal life) rather than the direct impact later in life [29,30].

**Figure 1:**
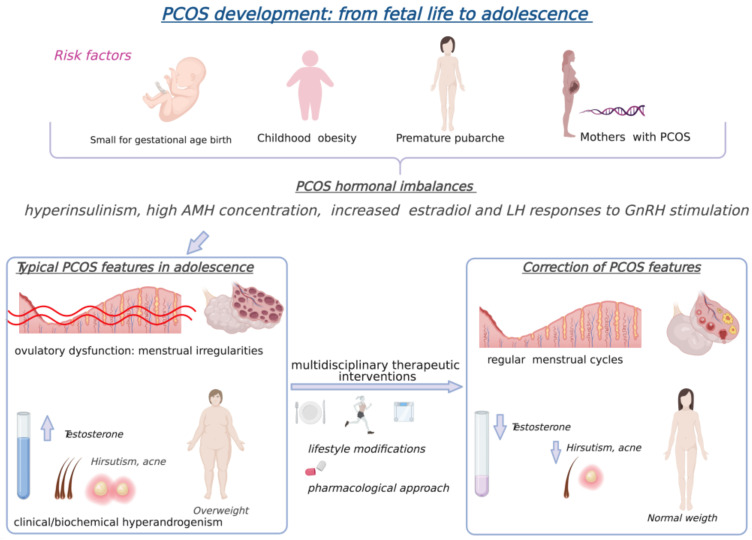
Polycystic ovary syndrome development: from fetal life to adolescence. Created with BioRender.com (accessed on 26 May 2021). AMH: anti-Müllerian hormone; LH: luteinizing hormone; GnRH: gonadotropin-releasing hormone.

Based on the studies performed on youth at risk of PCOS, the hypothetical model for PCOS pubertal ontogeny involves, firstly, the genetic predisposition to hyperinsulinism. Later in life (at adrenarche and at neuroendocrine puberty), this predisposition leads to increased androgen steroidogenesis in response to corticotropin and gonadotropin stimulation. Then, the hyperandrogenic environment promotes postpubertal neuroendocrine dysfunction, impairing the GnRH pulse generator and resulting in increased LH and decreased FSH release. These neuroendocrine imbalances support the progression to PCOS development, further enhancing hyperandrogenemia and ovulatory dysfunction [11] (Figure 1).

In adolescents, determining not only the etiology of PCOS but also the diagnostic criteria is a challenge, because the criteria used for diagnosis [31] in adulthood cannot be applied to adolescents, and because the criteria for diagnosis during adolescence have not been uniformly defined yet. 

Indeed, in recent years, three different sets of recommendations [17,18,19] for the diagnosis of PCOS in adolescence have been published. The guidelines agree on the essential features of this condition, but many details differ among the three [32].

The fundamental diagnostic criteria evidenced in the three documents are unexplained ovulatory dysfunction and clinical and/or biochemical evidence of hyperandrogenism [17,18,19].

Ovulatory dysfunction is defined as an abnormal menstrual pattern for age, but the menstrual abnormalities are not univocally described. Furthermore, due to anovulatory cycles commonly occurring after menarche, it is frequent to have menstrual irregularities. These include amenorrhea (primary or secondary), oligomenorrhea and excessive uterine bleeding [17].

Primary amenorrhea is defined as the absence of menarche at 15 years of age or by 3 years after the onset of breast development; secondary amenorrhea as the absence of a menstrual period for more than 90 days after previously menstruating; oligomenorrhea as an average cycle longer than 60 days (for first year post-menarche) or 45 days (for second and third years post-menarche); and excessive uterine bleeding as bleeding that occurs more frequently than every 21 days, is prolonged (lasting more than 7 days) or is heavy [32].

The guidelines do not agree on the timing of persistence of the menstrual irregularities after menarche—one [19] or two years [17,18]—but agree that following up those girls with menstrual irregularities in the cited period is necessary, as they are considered to be at risk of PCOS, eventually starting treatment to decrease future comorbidities regardless of the definitive diagnosis [33].

Regarding hyperandrogenism, the three guidelines consider hirsutism as clinical evidence of androgen excess. According to the latest criteria [19], mild hirsutism is sufficient clinical evidence of androgen excess, while the previous documents [17,18] consider moderate–severe hirsutism. Severe inflammatory acne, unresponsive to topical therapy, is also considered an indication for androgen levels testing according to the latest recommendations [19].

All guidelines recommend total and free testosterone measurement as a biomarker of hyperandrogenism and, eventually, further laboratory tests individualized to rule out other causes of virilization (such as non-classical congenital adrenal hyperplasia, Cushing’s or McCune–Albright syndrome and androgen-secreting tumors) according to the history and the other clinical features of the patient [17,33].

Moreover, the three international expert conferences agree that neither polycystic ovary morphology (and, thus, radiologic procedures to assess it [34]) nor obesity, insulin resistance or severe cystic acne—although common in these patients—alone can be considered diagnostic criteria in adolescents [18,20,33]. These additional clinical features and the assessment of biomarkers such as AMH or testosterone-to-dihydrotestosterone ratio (T/DHT) can be evaluated together with the required features, but should not be considered independently diagnostic [18,19], Table 1.

As PCOS is of great clinical and psychological concern [35] for adolescent girls with lifelong metabolic [36] and reproductive repercussions [3], early and patient-centered treatment is crucial [19]. 

Thus far, no pharmacological treatment has been approved in adolescents, but there are different options for coping with PCOS symptomatology, divided into baseline and additional treatments [18]. Among the baseline interventions, the first line of treatment is lifestyle modification, including diet [37], physical activity and weight loss [38]. These interventions have been shown to alter the course of disease in overweight and obese girls [3], decreasing androgen levels and menstrual irregularities and improving cardiometabolic health [18]. In girls of a normal weight, instead, physical activity correlated with a reduction in metabolic syndrome development but not weight loss alone [18]. As reported [38], vigorous aerobic exercise practiced consistently over the long term (at least three days per week for 30 min or more) and associated with heart rate and/or VO_2_max monitoring (≥60% VO_2_max) can improve insulin sensitivity. Improvements in androgen seem to be more likely with combined exercise that includes progressive resistance training or strength training three days per week on non-consecutive days [38].

Within the basic interventions, there is also local therapy and cosmetics, mainly used to ameliorate hirsutism and acne [5].

Among the additional treatments, the main pharmacological options are estrogen–progestin contraceptive pills, metformin and antiandrogens.

Combined estrogen–progestin therapy remains one of the main used therapies in PCOS patients; however, although found to be beneficial for hirsutism, acne and menstrual regulation, recommendations [18,19] have stated that additional high-level studies fail to approve its use and underline the best specific formulation to be chosen. 

Metformin is a common insulin-sensitizing agent and has been shown to be beneficial in obese/overweight girls with PCOS and in girls of normal weight with PCOS and hyperinsulinemia. Specifically, this agent promotes weight loss, menstrual cycle, acne and glycemic control [39]; thus, despite not being licensed for this syndrome, it is widely used. 

The antiandrogens used are of two types: androgen receptor blockers (such as spironolactone, flutamide and cyproterone acetate) and 5-alpha reductase inhibitors. There is no evidence that one type is preferable, but spironolactone is the most used in clinical practice [5]. Importantly, due to its potential teratogenicity, it is recommended to use it with oral contraceptives [5,18].

The guidelines also recommend, when possible, a combination approach, based on the association of previously cited therapies that act synergistically on different systems and improve PCOS clinical features and psychological issues [5].

This underlines the need for further studies in this field, especially in adolescence, since, as mentioned, still no pharmacological treatment has been approved [18]. The emerging role of glucagon-like peptide-1 (GLP-1) receptor agonists (GLP-1 RAs) as a therapeutic option for obese women with PCOS has been reported in adult age, and the benefits in adolescents could be considered [40,41].

## 4. Polycystic Ovary Syndrome and Obesity

Obesity is defined as a condition characterized by an excessive fat accumulation that has negative health consequences, being a risk for many diseases, including a wide spectrum of endocrine and reproductive disorders [42]. Although neither necessary nor sufficient for PCOS development, obesity—especially visceral obesity—amplifies and worsens all metabolic and reproductive outcomes in PCOS [43].

Obesity is extremely diffuse worldwide and represents a major public health problem in both childhood and adulthood [44,45]. In accordance with the World Health Organization data, the global prevalence of overweight and obesity in females aged 5-19 years has risen from 4% in 1975 to 18% in 2016 [46]. Given the high percentage of young reproductive-aged women affected, obesity is also particularly common in PCOS patients.

Up to 80% of women with PCOS in the United States and 30–50% in other countries are overweight or obese [9,47,48]. However, some authors state that this high percentage may be overestimated because of a referral bias, since it was calculated among women referred to reproductive endocrinologic clinics, whereas the prevalence of obesity among unselected women with PCOS is not far from that in the general population [49,50].

Nevertheless, these data remain consistent with the higher severity of PCOS presentation in the case of obesity, which likely results in a more frequent need for assessment in specific clinical settings. 

Numerous studies proved that women and adolescents with PCOS and obesity have more pronounced signs of hyperandrogenism and insulin resistance; higher BMI values positively correlated with free androgen index (FAI), total testosterone, free testosterone, homeostatic model assessment for insulin resistance (HOMA-IR), fasting insulin, fasting glucose, total cholesterol, low-density lipoprotein cholesterol (LDL-C), triglyceridemia, estradiol (E2) and androstenedione but negatively correlated with circulating levels of sex-hormone-binding globulin (SHBG), inhibin B and high-density lipoprotein cholesterol (HDL-C) [51,52]. Obese and overweight adolescents with PCOS also presented a higher frequency of hirsutism and acanthosis nigricans [53].

Community-based prevalence studies of PCOS in adolescents are scarce; nonetheless, it is reported that nearly 30% of adolescents with PCOS are overweight [54,55,56].

Diagnosing PCOS in adolescents is challenging because of the partial overlap between some signs of PCOS and changes during normal puberty—above all, menstrual irregularities and IR [26]. Menstrual disorders are commonly seen in the first to second postmenarcheal years and they are a sign of a not-yet-complete maturation of the hypothalamus–pituitary–gonadal axis [57]. The onset of puberty is associated with a physiological state of IR, a function to promote the rapid growth typical of this phase of development. There is a reduction in insulin sensitivity of up to about 30–50%, even in conditions of normal weight, compensated for by a significant increase in insulin secretion [49]^,^ [58]. In healthy children, this phenomenon usually appears at Tanner stage 2 and is self-limiting, with a gradual return to near pre-puberty levels of insulin sensitivity at the end of puberty. Nevertheless, during this period, the risk of developing conditions associated with IR is higher, including PCOS [49]. The clinical features of PCOS often begin during puberty, suggesting that the metabolic and reproductive changes associated with puberty may create fertile ground for triggering the pathophysiology of PCOS [26,59]. IR has a pivotal role in the genesis of the syndrome; it is often seen in patients affected by PCOS and it is exacerbated by obesity [9,60].

Moreover, a link emerged between obesity during adolescence and the appearance of PCOS later in life. A longitudinal population-based study pointed out that being overweight or obese at age 14 increases the risk of menstrual disorders and hirsutism in adulthood [61]. 

It should be underlined that obesity is usually classified according to body mass index (BMI). However, BMI has some limitations, mainly related to not providing an accurate index of body composition, nor of fat distribution [62]. Therefore, it may be particularly useful to measure waist circumference (WC) and calculate WC/height ratio (WC/H), which are effective indicators of abdominal obesity. WC/H > 0.5 is indicative of visceral obesity. This parameter is recognized as a better predictor of metabolic risk in youth [63].

A central distribution of body fat is more likely to characterize PCOS patients. Higher visceral adipose tissue (VAT) has been described even in lean women affected by the syndrome, and it may be associated with worse metabolic outcomes [64,65]. Evidence suggests that there are qualitative and quantitative dysfunctions of adipose tissue in PCOS [66], as discussed below.

### The Adipose Tissue and Ovarian Dysfunction

Adipose tissue is widely recognized as an active endocrine organ producing and secreting biologically active molecules called “adipokines”. Adipokines are involved in the regulation of various homeostatic processes such as energy metabolism, hunger and satiety regulation, insulin sensitivity, inflammation, atherosclerosis, cell proliferation and reproduction [67]. Adipose tissue cytokines include many molecules with pro-inflammatory activity (leptin, resistin, osteopontin, interleukin (IL)-6 and -10, tumor necrosis factor (TNF)-α, etc.) and some with anti-inflammatory action (such as adiponectin and omentin) [68].

In obesity, adipose tissue undergoes structural and functional changes, leading to a state of hyperinsulinemia, hyperlipidemia, hyperleptinemia and chronic low-grade inflammation [69]. Adipose tissue function is dysregulated and is characterized by hypertrophic adipocytes, due to an increased triglyceride content, impairment in adipokine secretion, a pro-inflammatory gene expression profile, immune cell infiltration and decreased insulin sensitivity. Although less well-outlined, it seems that similar alterations occur in the adipose tissue of patients with PCOS [70].

Obesity is one of the best known risk factors for IR, in adults as well as among adolescents [71]. It has been estimated that obese euglycemic individuals have around 30% reduced insulin sensitivity compared to euglycemic subjects of normal weight [72]. In insulin-resistant status, adipose tissue lipolysis is accelerated, and especially visceral adipocytes are more sensitive to lipolysis stimulated by catecholamines and less sensitive to the anti-lipolytic action of insulin. IR is indeed significantly related to visceral obesity, and visceral depot deserves attention because it is considered to be more metabolically active and because it releases factors to the portal venous system and, thus, may directly have an impact on the liver [73]. Alterations in lipolysis are also described in PCOS. Ek and co-authors reported a marked increase in catecholamine-induced adipocyte lipolysis in PCOS women and a significant correlation between fat cell size and lipolytic responsiveness [74].

Excessive free fatty acids may exert a toxic effect in reproductive tissues, producing persistent cell damage and a chronic low-grade inflammatory state [75]. It is hypothesized that obesity alters the mitochondrial function in the oocyte through lipotoxicity.

The lipotoxicity theory implies that lipids stored in adipocytes in the form of triglyceride are biologically inert, and that metabolic dysfunctions are primarily due to the increased exposure of the cells to fatty acids when the excessive fatty acids overwhelm the storage capacity of adipose tissue and free fatty acids accumulate in ectopic sites. This exposure may result in cell dysfunction, cell death and inflammation [76]. Furthermore, higher levels of circulating free fatty acids damage non-adipose cells by increasing reactive oxygen species (ROS) that, in turn, induce mitochondrial stress and apoptosis of multiple cell types, including oocytes [75].

Enlarged adipocyte size was observed in women with PCOS. The same study described lower serum adiponectin in patients with PCOS and a strongly positive association between adiponectin and insulin sensitivity [77].

Adiponectin is an abundant plasmatic adipokine, mainly produced and secreted by white adipose tissue and widely known to have anti-inflammatory, anti-atherogenic and insulin-sensitizing properties, through which it shows protective effects in various processes such as inflammation and energy metabolism [67]. Adiponectin plasma concentration correlates with the adipose tissue level [78]; in obese compared to control patients, adiponectin concentrations have consistently been found to be abnormally low [79,80].

In recent years, the effects of adiponectin in the modulation of reproductive functions have been increasingly studied. In vitro and in vivo evidence suggests that it may be included among hormones that control the interactions between the body’s energy homeostasis and fertility [78]. In females, it controls ovarian steroidogenesis and folliculogenesis [81]. As stated by Drolet and co-authors, there may be differences in adiponectin release between subcutaneous and visceral fat [82]. They tested subcutaneous and omental adipocytes of women undergoing abdominal hysterectomy, showing that the greatest reduction in protein secretion in women with obesity affected visceral adipocytes. Furthermore, an inverse correlation was noted between the size of visceral adipocytes and the amount of adiponectin released. This could be the main determinant of the hypoadiponectinemia characteristic of obesity. Weight loss is correlated with an increase in circulating adiponectin, suggesting a possible functional recovery of adipocytes [83].

A systematic review and meta-analysis confirmed that women with PCOS have lower levels of adiponectin. Lower adiponectin values were associated with the IR observed in women with PCOS compared with controls [84]. Since adiponectin exhibits insulin-sensitizing properties, reduced levels of adiponectin may contribute to systemic insulin resistance in women with PCOS [70].

Obesity is associated with a chronic inflammatory state. Immune cells, primarily macrophages, infiltrate adipose tissue, promoting both adipocyte hypertrophy and cytokine release [73]. In addition to being more present, the adipose tissue macrophages of obese individuals undergo a phenotypic switch and secrete pro-inflammatory cytokines (TNF-α, IL-6 and IL-1β) [69]. The activated macrophages infiltrate other organs and may play a role in the development of obesity-induced insulin resistance. A study by Mannerås-Holm and co-authors found similar adipose tissue macrophage densities in the PCOS group versus controls [77].

It is, therefore, a complex and integrated frame, in which the interaction of metabolic, hormonal and immune stimuli, altered in obesity status, could act on the reproductive axis, explaining the high prevalence of reproductive disorders in adolescents and women with obesity, including PCOS.

## 5. Polycystic Ovary Syndrome and Insulin Resistance

IR is defined clinically as the inability of a known quantity of exogenous or endogenous insulin to increase glucose uptake and utilization in an individual as much as it does in a normal population [85]. In an insulin-resistant status, peripheral tissues have less sensitivity to the action of insulin; therefore, higher concentrations of the hormone than normal are required to maintain normoglycemia, resulting in the development of compensatory hyperinsulinemia [86].

**Figure 2:**
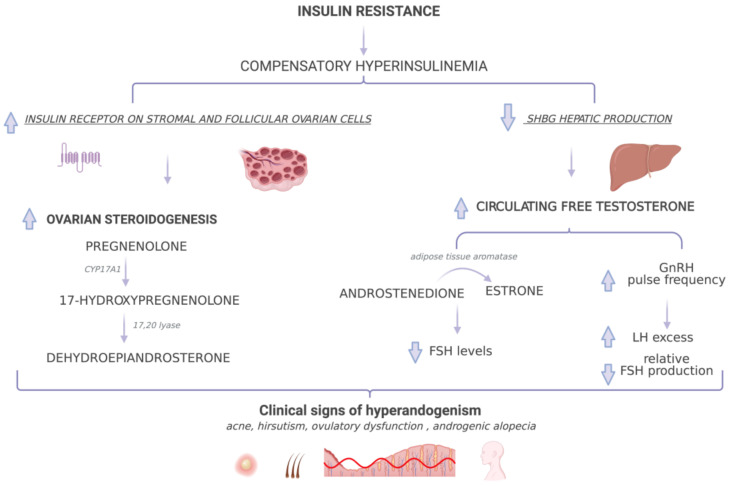
Insulin resistance and development of polycystic ovary syndrome. Created with BioRender.com (accessed on 26 May 2021). FSH: follicle-stimulating hormone; LH: luteinizing hormone; GnRH: gonadotropin-releasing hormone; SHBG: sex-hormone-binding globulin. FSH: follicle-stimulating hormone; LH: luteinizing hormone; GnRH: gonadotropin-releasing hormone; SHBG: sex-hormone-binding globulin.

Although not invariably present in PCOS [87], IR plays a central role in its pathogenesis, and it is a common finding in subjects with PCOS, both lean and affected by obesity [88] (Figure 2).

Between 44% and 70% of women with PCOS are insulin-resistant [10], and women with PCOS and obesity are more insulin-resistant than obese controls [89]. These data suggest that both obesity and PCOS influence insulin sensitivity and that their coexistence may increase IR. DeUgarte and co-authors studied the prevalence of IR in a large population of unselected patients with PCOS (271 subjects, mean age 27.4 ± 7.5 years) using the homeostatic model assessment for insulin resistance (HOMA-IR) and percent β-cell function (HOMA-% β- cell): 64.4% of PCOS patients were insulin-resistant, and a positive correlation of HOMA-IR and HOMA-% β- cell with BMI was found among the PCOS group. Notably, patients with IR had higher BMIs, with a prevalent visceral fat distribution; a higher excess of androgens; and more severe clinical PCOS manifestations, such as hirsutism, acne and ovulatory dysfunction, compared to PCOS women without IR [90]. 

PCOS is a heterogeneous disorder, and the four different recognized phenotypes differ not only for reproductive features but also for metabolic ones. Insulin resistance frequency differs among subgroups, with the classic phenotype (oligo/anovulation + hyperandrogenism + PCOM) showing the highest prevalence of IR, followed by the ovulatory phenotype (hyperandrogenism + polycystic ovarian morphology (PCOM)) [87]. Conversely, the PCOS phenotype characterized by anovulation and PCOS with normal androgen levels most often shows normal insulin sensitivity [10,87,91]. Overall, these findings suggest that hyperandrogenism may have a major impact on insulin sensitivity, although none of the PCOS diagnostic features per se are invariably associated with IR [87]. It should be noted that some authors described that even PCOS patients without overt hyperandrogenism showed gonadotropin derangement and had lower mean sex-hormone-binding globulin (SHBG) values than controls did, a finding that suggests abnormalities in insulin levels, probably related to the higher waist circumference values of this PCOS group compared to controls [92].

Determining the prevalence of IR among adolescents with PCOS is more challenging, because puberty itself is associated with a physiological state of IR [86]. However, IR and hyperinsulinemia appear early in the disorder [88], which was demonstrated in a study by Lewy and co-authors in a group of obese girls with PCOS (mean age 12 ± 0.7 years). The study findings indicated that adolescent girls with POCS and obesity had around a 50% decrease in peripheral insulin sensitivity, evidence of hepatic insulin resistance and compensatory hyperinsulinemia compared to non-PCOS girls with obesity (control group) [93]. Indeed, the β-pancreatic cells of adolescent girls with PCOS are able to respond to IR with increased insulin secretion [93]. The body is therefore exposed to higher circulating insulin levels than normal, and this may have consequences in the ovaries, where insulin functions have a co-gonadotropin [94].

Decreased peripheral insulin sensibility was also described in normal-weight girls with PCOS, although not as impaired as in girls with PCOS and obesity [95]. The more consistent presence and severity of IR and hyperandrogenism in adolescents with PCOS and obesity compared to the PCOS group without obesity and to the control group were also confirmed in a study carried out by Zhao and co-authors in Chinese adolescent girls [96]. Therefore, IR is certainly observed in PCOS adolescents, and obesity may have an additive effect. The authors also reported an earlier onset of menarche in adolescents affected by PCOS.

In addition to the extensively known metabolic effects, insulin is involved in controlling growth processes (mitogenic action) and plays a role in ovarian physiology and pathophysiology [85,94,97].

Insulin receptors are widely distributed in both stromal and follicular ovarian cells, where the hormone participates in follicular development and ovarian steroidogenesis [97].

Hyperinsulinemia leads to increased androgen production, which, in turn, may impair insulin sensitivity [98]. This vicious cycle induces and worsens the reproductive and metabolic abnormalities that characterize PCOS. 

IR and the resulting compensatory hyperinsulinism are linked to hyperandrogenemia in various ways. Firstly, insulin promotes androgen synthesis from theca cells, either directly or by enhancing the response of theca cells to circulating LH by induction of steroidogenic enzymes [99]. Theca cells are the predominant site of androgen production in the ovaries, and insulin acts on theca cells mainly via its own receptor. It seems that the theca cells of women with PCOS are hyperresponsive to the androgen-stimulating action of insulin [10] and have increased expression of some steroidogenic enzymes. In particular, a study that analyzed cytochrome P450 17α-hydroxylase (CYP17A1) expression in PCOS theca cells found an increased expression, both at baseline and after combined treatment with insulin and LH [100]. CYP17A1 is an enzyme with both 17αhydroxylase and 17,20-lyase activities and mediates the conversion of pregnenolone by 17-hydroxylation to synthesize 17-hydroxypregnenolone, which is transformed by 17,20-lyase activity to dehydroepiandrosterone (DHEA) [101].

Secondly, insulin decreases hepatic production of SHBG, the plasmatic carrier of sex steroids, thus increasing the amount of circulating free testosterone, which is responsible for the clinical signs of hyperandrogenism (hirsutism, acne and alopecia). Moreover, testosterone is incompletely aromatized by granulosa cells because of relative FSH deficiency [10].

Excessive circulating levels of testosterone and androstenedione may undergo extraglandular aromatization to estradiol and estrone, respectively. Adipose tissue has an aromatase capable of transforming androstenedione to estrone, and the amount of androstenedione converted to estrone increases in proportion to obesity and aging [102]. Therefore, obesity impacts the biochemical levels of circulating sex hormones.

Moreover, high circulating estrone may contribute to impaired gonadotropin secretion. The administration of estrone benzoate in women with PCOS progressively reduced FSH levels without altering LH, thus intensifying the disparity of gonadotropin secretion typical of the syndrome [103]. Data support the hypothesis that FSH release impairment by chronic acyclic estrogen production, to which non-glandular aromatization of circulating androgen contributes, causes, to a large extent, anovulation in PCOS.

It is hypothesized that testosterone may antagonize progesterone negative feedback on GnRH, thus increasing the GnRH pulse frequency instead of allowing progesterone to slow the GnRH pulse, according to the model proposed by McCartney in girls with obesity [104]. Androgen-related increases in GnRH pulse frequency foster LH excess and relatively low FSH production [26]. 

Insulin signaling in the central nervous system appears to be critical for ovulation. Hyperinsulinemia may interfere with GnRH signaling, promoting increases in LH and GnRH pulse frequency and amplitude [99]. Disruption of the delicate equilibrium between gonadotropins at the hypothalamus and pituitary levels leads to ovarian dysfunction. Obesity may have an additive effect on neuroendocrine disruption, considering that it is associated with low-grade inflammation that may also be present in the central nervous system. For instance, evidence suggests that in obesity, hypothalamic microglia are probably activated by an increased circulating level of saturated fatty acids [69]. 

Growing evidence suggests that lipotoxicity, which is the excessive exposure of non-adipose tissues to free fatty acids (FFAs), causing numerous functional cellular defects, plays a role in the development of peripheral and hepatic insulin resistance and β-cell dysfunction. Higher levels of FFAs damage non-adipose cells by increasing ROS that, in turn, induce mitochondrial stress and apoptosis of multiple cell types, including oocytes [75]. Mitochondrial dysfunction was also described in PCOS [86]. High levels of FFAs may result either from the uptake of circulating FFAs, which are higher during fasting, or from accelerated adipose tissue lipolysis, which happens in insulin resistance, where the metabolic anti-lipolytic action of insulin is compromised. Though few in number, some studies have described an increase in fasting circulating FFA in adolescents and women with PCOS [105]. Some authors suppose that another contribution of excessive androgen production to PCOS may derive from increased androgen secretion from ovarian and adrenal cells in response to an overexposure of these tissues to circulating FFAs [106,107].

Insulin seems to also have effects on adrenal androgen production. Androgens are synthetized in the ovaries and adrenal glands in females. Clinically, the measurement of circulating levels of dehydroepiandrosterone sulfate (DHEAS) is traditionally used as a marker for adrenal androgen excess, since 97–99% of this hormone is secreted by the adrenocortical zona reticularis [108]. Even though PCOS is primarily an ovarian disease with increased androgen production, adrenal androgen excess is also common, and around 20–30% of women with PCOS have elevated levels of dehydroepiandrosterone (DHEA) and DHEAS. Some patients with PCOS even have isolated increases in serum DHEAS [10,108]. The adrenal glands of some women with PCOS show exaggerated adrenal steroidogenesis in response to adrenocorticotrophic hormone (ACTH) [109].

Insulin also appears to potentiate basal and ACTH-stimulated adrenal androgen production [26]. It is reasonable to think that DHEAS levels are associated with IR in women with PCOS because they decrease with insulin-sensitizing treatment [9].

In conclusion, although IR appears to be partly independent of obesity, concomitant obesity exacerbates insulin resistance and hyperandrogenemia in PCOS. In a considerable proportion of PCOS patients, the intrinsic defect in steroidogenic activity and androgen secretion is triggered or enhanced by factors such as hyperinsulinism. Hyperinsulinism may be the consequence of IR and may derive from the production of metabolically active molecules by visceral adipose tissue, because these factors may facilitate ovarian and adrenal androgen synthesis of predisposed subjects [8].

Despite this high prevalence, obesity and IR are not part of the required diagnostic criteria for PCOS. However, they raise the risk of metabolic complications such as metabolic syndrome, type 2 diabetes mellitus and dyslipidemia in adolescents and women with PCOS [54].

### Molecular Mechanism of Insulin Resistance

Growing evidence suggests that IR in PCOS is due to a post-receptor defect in insulin signaling and that it is selective. It seems to selectively affect the metabolic pathway of insulin action but not the other actions, and specifically not the mitogenic pathway [85,110].

Insulin acts by binding to the extracellular domain of its cell surface receptor. Insulin receptors are heterodimers formed by two α subunits and two β subunits. Their structure consists of an extracellular and an intracellular portion, the latter being endowed with tyrosine kinase activity. Insulin binding to the receptor induces autophosphorylation of the β subunit on specific tyrosine residues, triggering a complex intracellular cascade [111]. The activated insulin receptor then phosphorylates intracellular substrates, including insulin receptor substrates (IRS 1–4) [10].

There are two main downstream signaling pathways: the metabolic and the mitogenic pathway. The former involves kinases such as phosphatidylinositol 3-kinase (PI3-K) and the serine/threonine kinases Akt/protein kinase B (PKB), and is also known as the PI3-K pathway. Through the mentioned pathway, insulin stimulates cellular glucose internalization by promoting the translocation of glucose transporter 4 (GLUT4) [112]. The latter, also known as the MAPK/ERK pathway, begins with the phosphorylation of Shc and primarily mediates cell growth and steroidogenic insulin effects [101,113].

Nowadays, there is agreement that the metabolic pathway is disrupted in PCOS, without involving the mitogenic cascade. Therefore, IR can be selective and can affect only the metabolic actions of insulin [10,85,114]. Conversely, the MAPK/ERK pathway appears to be preserved, if not activated, by the hyperinsulinemic state [115]. A recent experimental study confirmed the constitutive activation of the MAPK/ERK pathway in the skeletal muscles of some women with PCOS [116].

Evidence suggests that the main pathophysiological mechanism involved in the defect is the significantly increased serine phosphorylation of the insulin receptor and insulin receptor substrate-1 (IRS-1). Serine phosphorylation may inhibit regular receptor signaling and provoke a diminished insulin-mediated activation of PI3-K [117]. The mentioned abnormality was described in cultured skin fibroblasts, skeletal muscle biopsies and adipocytes of PCOS patients [117,118].

A hypothesis that may explain this abnormal phosphorylation pattern involves either a serine kinase external to the insulin receptor responsible for the phosphorylation, or the kinases that constitute the MAPK/ERK pathway, whose constitutive activation may also contribute to the serine phosphorylation of insulin receptor substrate (IRS)-1 [10,117].

Notably, studies have reported that treatment with serine kinase inhibitors corrects the phosphorylation defect, at least in fibroblasts from PCOS patients [119]. Moreover, pharmacological inhibition of MEK1/2 suppresses MAPK-ERK1/2 activation, reduces IRS-1 serine 312 phosphorylation and augments IRS-1-associated PI3-K activation [120].

These findings suggest the role of both a serine kinase external to the insulin receptor and the constitutive activation of the MAPK/ERK pathway, which impairs metabolic signaling in PCOS via serine phosphorylation of IRS-1/insulin receptor.

The defect in insulin receptor signaling in PCOS seems to be intrinsic and independent of obesity and type 2 diabetes mellitus [118]. However, it may be possible that these abnormalities confer increased susceptibility to circulating factors that induce insulin resistance, such as free fatty acids or TNF-α. [10]. It is well recognized that obesity is a state of chronic low-grade inflammation, characterized by aberrant pro-inflammatory cytokines and adipokine secretion, including TNF-α. The molecular mechanisms by which fatty acids, inflammatory cytokines and adipokines interfere with insulin are not fully known, but it is believed that they operate at the post-receptor level, and the interference with phosphorylation may be an intriguing speculation that deserves further detailed analysis.

In addition, the possible role of mitochondrial dysfunction in the development of IR in PCOS has also been mentioned previously in discussions, as well as the fact that mitochondrial dysfunction also appears in obesity.

Lastly, it has also been described that the serine phosphorylation of P450c17, a central enzyme involved in ovarian and adrenal steroidogenesis with both 17α-hydroxylase and 17,20-lyase actions, increases its activity [121]. The consequence is increased androgen synthesis. As stated by Diamanti and Dunaif, it has, thus, been speculated that the same kinase or factor may inhibit insulin signaling (through the serine phosphorylation of insulin receptor and IRS-1) and, at the same time, increase androgen production in PCOS [10]. This intriguing hypothesis creates a direct link between IR and hyperandrogenism. However, despite several attempts, the possible common factor that phosphorylates both IRS-1/insulin receptor and P450c17 has not yet been found [10].

## 6. Medical Nutrition Therapy for Polycystic Ovary Syndrome

### 6.1. Role and Mechanisms of Action of Dietary Macronutrients

Treatment for adolescents and young women with PCOS includes lifestyle changes, dietary interventions and weight loss as needed, in addition to medications.

The approach of MNT in these patients aims at improving insulin resistance and metabolic and reproductive functions by means of a personalized diet, considering energy restriction if weight loss is needed as well as the nutrient composition of the diet, which, regardless of weight loss, affects insulin sensitivity.

Given the prevalence of overweight, obesity and insulin resistance, a relatively low-energy diet, promoting from 5% to 15% reduction in weight, can improve insulin resistance, androgen concentration, reproductive system dysfunctions and fertility in these women [122].

Furthermore, diet composition, regardless of weight loss, affects insulin sensitivity. An important role is played by the quality and quantity of carbohydrates introduced with the diet; in fact, reduction in the glycemic load leads to a reduction in postprandial glucose levels and consequent hyperinsulinemia. A diet rich in complex carbohydrates, especially from unrefined foods, and fiber has been linked to greater insulin sensitivity [123]. Dietary fiber, especially the soluble type, has been shown to result in gastric emptying delay; digestion and absorption of nutrients, such as glucose; and increased satiety [124].

Foods derived from plant sources not only provide dietary fiber, promoting glycemic control, but are also sources of phytochemicals such as polyphenols, which decrease hyperglycemia and improve acute insulin secretion and insulin sensitivity [125]. Even more remarkable is that diets with a high fiber content exert a protective role in the pathogenesis of metabolic disease due to the presence of inositol(s) as causative protective agents. Indeed, myo-inositol (MI) and its phosphate derivatives have demonstrated numerous valuable health effects, including anti-diabetic ones [126], gaining considerable interest in practical applications as supplements. Combined administration of myo-inositol (MI) and d-chiro-inositol (DCI) with an MI:DCI ratio of 40:1 was observed to be a valid treatment for IR in women with PCOS, since inositol isomers show activities that mimic those of insulin [127].

Moreover, diets rich in fiber have been shown to interact directly with gut microbes, impacting gut microbiota composition, diversity and richness. Many studies have investigated the relationship between gut microbiota disorders and PCOS, suggesting that the gut microbiota is involved in the development of IR and menstrual disorders in PCOS patients by affecting intestinal wall permeability [128]. A low-carbohydrate, high-fiber diet increases short-chain fatty acids (SCFAs) (acetate, propionate and butyrate) produced by the gut microbiota, maintaining the integrity of the intestinal barrier and reducing the risk of chronic sub-clinical inflammation.

Butyrate, in particular, mainly produced by Firmicutes, appears to prevent the development of IR and obesity, as opposed to a diet rich in simple sugars and refined complex carbohydrates, which triggers inflammation, IR, androgen production and dysbiosis [129,130].

Gut microbiota (GM) regulation should, therefore, be considered as a potential adjuvant clinical treatment for PCOS based on treating the GM to provide new ideas for clinical treatment [131].

The use of probiotics and synbiotics in women with PCOS improved some hormonal (FAI and SHBG) and inflammatory (nitric oxide and malonyl dialdehyde) indices [132], suggesting that probiotics as well as prebiotics may be used to treat metabolic abnormalities associated with PCOS. 

On the other hand, excessive intake of animal-based foods leads the body’s acid base balance towards acidosis, which has been linked to insulin resistance and reduced glucose homeostasis [133].

Moreover, animal-based foods, rich in saturated fats, are associated with reduced insulin sensitivity, assisting pancreatic beta cell dysfunction and death [134].

However, it is well known that not all fats from animal sources are equal. Positive effects on IR have been associated with omega-3 fatty acids, particularly eicosapentaenoic acid (EPA) and docosahexaenoic acid (DHA). The most well-known sources of omega-3 fatty acids include fatty fish such as salmon, tuna and sardines; however, plant-based foods, such as nuts and some seeds, contain alpha-linolenic acid, the precursor of EPA and DHA. In women with PCOS, supplementation with omega-3 fatty acids, α-lipoic acid and N-acetylcysteine has shown anti-inflammatory and antioxidant effects and insulin sensitivity improvement [135]. 

### 6.2. Role and Mechanisms of Action of Dietary Micronutrients

Inadequate intake and subsequent deficiencies of zinc, magnesium and selenium are involved in decreased secretion and/or activity of insulin, while their supplementation, in both diabetic and non-diabetic subjects, has shown HOMA-IR and fasting glucose improvements [136]. Both zinc and selenium are essential micronutrients for metabolism and regulate several enzymes involved in the production and neutralization of ROS [136]; therefore, they act as antioxidants counteracting the oxidative stress involved in PCOS and are probably significant in its pathogenesis [137]. Additionally, chromium (III) supplementation has been shown to effectively improve glucose tolerance by reducing IR and improving insulin-binding capacity and the number of receptors and insulin receptor enzymes by increasing insulin sensitivity, beta cell sensitivity and insulin internalization [136]. Although there is still insufficient evidence to justify including chromium as a standard in the treatment of IR [138], randomized controlled trials (RCTs), such as the one conducted in 2016 that focused on chromium picolinate in women with PCOS, are providing solid grounds for effective ovulation stimulation and insulin resistance reduction [139].

In addition to minerals, vitamins also play a primary role. It is well known that vitamin D impacts insulin signaling and release, improves beta cell function and reduces IR [140]. PCOS patients supplemented with a continuous low dose of vitamin D (<4000 IU/day) showed an improvement in insulin sensitivity with regard to fasting glucose concentration and HOMA-IR [141]. Vitamin-D-deficient women with PCOS supplemented with calcium and vitamin D for eight weeks showed improved serum insulin levels and HOMA-IR [142]. Vitamin D supplementation also appears to be useful for increasing antioxidant capacity [140]; however, the current literature shows controversial findings regarding the ability of cholecalciferol to prevent or ameliorate oxidative stress biomarkers, and there is a need for further, high-quality studies testing the antioxidant effect of vitamin D supplementation [143].

Another group of important vitamins is B vitamins, particularly B12 and folate, which are important co-factors in the homocysteine/methionine process and in DNA repair. Vitamin B12 and folate deficiency can be linked to greater homocysteine and insulin resistance as well as higher risk of type 2 diabetes. High homocysteine levels are often observed in women with PCOS, and folate is a promising supplement to decrease the homocysteine levels in PCOS patients; however, further randomized controlled trials are needed to confirm this assumption [144]. 

### 6.3. Diets with Beneficial Effects on Insulin Resistance

PCOS symptoms and overall risk of developing long-term health problems can be greatly improved by losing excessive weight. Diet composition is also important, as reported above, and dietary modifications are recommended to improve the metabolic status. However, women with PCOS are usually non-compliant to long-term dietary recommendations, with scarce improvement in metabolic and reproductive outcomes [145]. 

Women with PCOS tend to underreport their dietary intake, which may be an obstacle to providing the effective nutritional counselling necessary to achieve meaningful clinical outcomes [145].

Recommendations for women with PCOS reflect previous observations regarding the beneficial effects of low-fat diets and the negative impact of high-carbohydrate diets [146]. Dietary changes recommended include balancing energy intake, limiting saturated and trans fats and shifting toward consumption of unsaturated fats, increasing intake of fruits and vegetables and limiting the intake of simple sugars and salt, which encompass the current concept of a “healthy diet” [147]. Such options are intrinsic to the eating habits of certain regional diets, such as the Mediterranean diet (MD), a plant-based diet poor in animal source foods and processed foods that is characterized by a high consumption of extra virgin olive oil (EVO), nuts and legumes and that has been associated with an improvement in diabetes risk and IR in subjects with PCOS and excessive body weight [148].

Adherence to an MD is associated with a reduction in inflammation as well as downregulation of cellular and humoral immunological pathways related to disease activity and progression [149].

Additionally, the MD is a low-glycemic-load diet that is rich in antioxidants—potential oxidative stress reducers [150].

The MD also includes lifestyle considerations such as engaging in regular physical activity, which has been reported to improve IR in women with PCOS [151]. Women with PCOS and excessive body weight who performed physical activity consistently for at least 12 weeks experienced a significant reduction in central adiposity and insulin sensitivity [151], revealing further beneficial effects on a number of health measures, including glycemic control [152].

Since most women with PCOS show marked compensatory hyperinsulinemia after intake of complex carbohydrates, there may be explicit benefits of diets with a low glycemic index/glycemic load (GL) in this group. These diets are more likely to improve IR in women with PCOS compared to a conventional healthy diet [153]. Women on a low glycemic index diet experienced changes in the number of ovulatory cycles, which could be related to a decrease in circulating serum androgen levels secondary to an improvement in IR [154], achieved by those with excessive body weight and attained with a weight loss of 4–5%.

Nevertheless, glycemic response also depends on the total intake of dietary carbohydrate, leading to the concept of GL, which represents both the quality and quantity of carbohydrate intake [155].

The GL of a meal varies according to the amount of fiber and protein in the meal and the processing techniques/cooking methods of the foods and significantly influences glycemic control [155], but the benefit in pursuing such a diet without considering energy intake and total carbohydrate intake in these patients should be further investigated [155].

This is why previous research has focused on low-carbohydrate diets, with less than 200 g of carbohydrates per day or less than 30% of daily energy intake from carbohydrates.

Low-carbohydrate diets have been demonstrated to effectively facilitate the treatment of infertility in obese PCOS patients due to an immediate positive impact on blood glucose levels, lipid levels, BMI, HOMA-IR, T, FSH and SHBG [154].

Recent studies have shown that effective weight loss and improvements in insulin resistance can also be achieved by means of a low-carbohydrate ketogenic diet (LCKD) in women with PCOS [156,157].

Ketogenic diets are characterized by a reduction in carbohydrates (usually to less than 50 g/day) and a relative increase in the proportion of proteins and fats. In an LCKD, 75% of the daily energy intake is derived from fat, 20% from protein and 5% from carbohydrates [158]. An LCKD is not a high-protein diet, but is usually high in fats, adequate in protein and low in carbohydrates.

Such a diet is considered absolutely safe for short cycles in women with PCOS, although there is no evidence of long-term side effects; the LCKD preserves the lean body mass thanks to a sufficiently adequate supply of dietary protein intake. Women with PCOS experienced a reduction in body weight, blood glucose and insulin levels after 12 weeks on such a diet, correcting hyperinsulinemia and HOMA-IR [159] and, consequently, improving body composition. Results after a 6-month period on an LCKD in women with PCOS and obesity showed a significant improvement in fasting insulin, which appeared to be the cause of LH/FSH ratio enhancement [156].

It is, however, worth noting the high dropout rate among participants and the mixed results on long-term efficacy [160]. In fact, some studies have shown an almost total weight regain after 2–3 years; nevertheless, the LCKD can be considered a safe nutritional approach considering that any dietary treatment has to be personalized. The LCKD is suitable for those who have already failed many weight loss dietary treatments in the past or for those who need to lose weight in the short term, but once they have reached the expected weight loss, it is advisable to shift to a balanced dietary pattern, such as the MD, together with physical activity to consolidate the results achieved in the long term [161].

**Figure 3:**
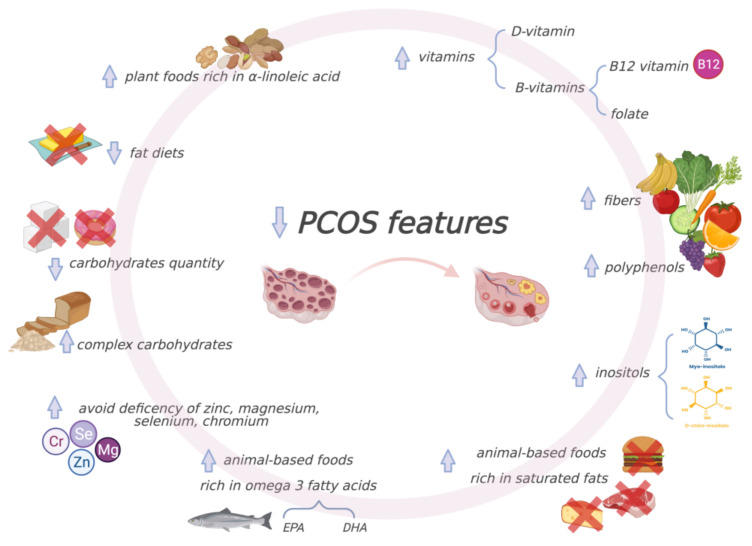
Macro- and micronutrients with benefits in polycystic ovary syndrome. Created with BioRender.com (accessed on 26 May 2021). EPA: eicosapentaenoic; DHA: Docosahexaenoic acid; ↑high; ↓low.

Many studies have shown the benefits of weight loss and lifestyle changes for improving PCOS signs and symptoms, also considering that women with PCOS have a greater risk of cardiovascular disease than women of the same age without the condition [162]. Therefore, nutritional assessment, eating habits and cultural background have to be considered in order to choose the best MNT by means of a personalized diet with low glycemic load, anti-inflammatory properties and energy restriction for weight loss, supplemented with micronutrients if needed, along with adequate nutritional counselling aimed at healthy lifestyle changes, including physical activity (Figure 3).

It is also worth bearing in mind that compliance with low-carbohydrate diets can be difficult for long periods, especially in those with a strong Mediterranean dietary cultural background, and that multidisciplinary treatment is mandatory.

## 7. Food Supplements in Insulin Resistance

The use of food supplements, whether present in food as bioactive molecules or administered as supplements, is increasing as a therapeutic approach to obesity and metabolic syndrome (MetS) [163]. Adolescence in particular requires special attention, both due to the need to optimize cardiovascular prevention in order to counteract cardiovascular risk and for growth requirements. According to the EFSA [164], food supplements are “concentrated sources of nutrients (or other substances) with a nutritional or physiological effect”. In recent years, several functional foods, bioactive compounds or nutraceuticals have been investigated and included for the treatment of various diseases [165].

Among them, inositol [166,167] has been shown to provide benefits for subjects with PCOS, improving IR and reducing the secretion of triglycerides.

Probiotics and prebiotics have also shown positive effects by modifying the gut microbiota, modulating the absorption of specific nutrients and improving metabolic outcomes. Other supplements, particularly red yeast rice, DHA, berberine and curcumin, have proven to be useful in PCOS treatment by means of IR improvement [168]. Although randomized controlled trials on the use of such compounds in pediatrics are scarce and mostly conducted on small sample sizes, particularly in children with MetS or PCOS, clinical benefits on lipid and glucose balance have been reported in both the short and medium term [169]. Of note, some of these supplements are not commonly used, and a limited number of studies have been conducted to prove their efficacy; however, they have been reported as potentially interesting treatments to be explored in future studies.

### 7.1. D-Chiro-f (DCI)

D-chiro-inositol (DCI) and myo-inositol (MI) are two stereoisomers of inositol that are easily found in plant-based diets and considered insulin sensitizers [170].

DCI presence depends on the activity of an epimerase able to convert MI into DCI. As soon as IR is manifested, the conversion rate is altered, with a consequent reduction in DCI intracellular concentration. Once DCI is optimized by means of supplementation, it positively impacts insulin receptor activity and, consequently, glucose metabolism [171]. Although there are two forms of inositol involved, DCI seems to be more effective in re-distinguishing correct insulin sensitivity and glycogen synthesis [170].

In animal models, DCI supplementation has shown significant molecular improvements, including cellular signaling pathways involved in IR (via PI3K/protein kinase B), glucose conversion to ATP and GLUT4 expression in skeletal muscle. Furthermore, DCI supplementation has shown a higher impact on IR and hyperglycemia in mice with type 2 diabetes (T2D), compared to MI [172,173,174].

On the other hand, a trial in women with PCOS supplemented with DCI at different dosages concluded that 500 mg/day for 12 weeks improves insulin sensitivity in those affected by obesity, while 1200 mg/day for 6 weeks was associated with an increased release of DCI-IPG mediator, enhancing insulin sensitivity [175].

In a study conducted on 11 children with obesity, ranging between 7 and 15 years of age, supplemented with inositol for 6 months (MI 1100 mg + DCI 27.6 mg + folic acid 400 μg), a greater reduction in fasting insulin as well as both blood glucose and insulin after 120 min was observed than in the control group [176].

Moreover, improved ovulation function, blood pressure and plasma TG concentrations were reported in another trial on 44 women with PCOS and obesity, supplemented with 1200 mg/day DCI for 8 weeks [176]. Finally, as PCOS is characterized by systemic silent inflammation due to increased oxidative stress, DCI supplementation (1000 mg/day) led to decreased ROS production in follicular fluid and a consequent reduction in IR [177,178].

### 7.2. Docosahexaenoic Acid (DHA)

Docosahexaenoic acid (DHA) is an ω-3 long-chain polyunsaturated fatty acid (LC-PUFA) belonging to the essential fatty acids since they cannot be synthesized ex novo by humans. 

DHA is particularly important for fetal brain development, optimal development of cognitive abilities and visual acuity [179].

Considering that PCOS is characterized by IR and, frequently, metabolic syndrome development with an increased risk of cardiovascular and metabolic disease, the role of dietary fats has been investigated.

High dietary levels of LC-PUFAs in relation to their ability to modulate inflammation can prevent the development of the main comorbidities associated with obesity, particularly MetS [180]. Low LC-PUFA levels, especially DHA, and high ω-6/ω-3 ratio in skeletal muscle membrane phospholipids have been associated with IR in adults [180]. 

Recent data suggest that DHA synthesis differs between children with severe obesity and children of normal weight. In a case–control study, children with the highest quartile of body mass index z-score (BMI z-score) showed higher plasma levels of ω-6/ ω-3 LC-PUFA. In addition, BMI z-score was negatively associated with plasma levels of DHA and, therefore, related to the degree of obesity [181]. Another study of 57 obese children with normal lipidemia, aged between 8 and 13 years, reported, after a year of nutritional intervention, that obese children had a reduced plasma level of saturated fatty acids and an increased concentration of monounsaturated fatty acids, PUFA ω-6, ω-3, arachidonic acid (AA), DHA and DHA/AA ratio [182].

A randomized controlled clinical trial (RCT) performed on 60 children with NAFLD showed that supplementation with DHA (both 250 and 500 mg/day) reduced liver steatosis and improved the HOMA-IR index [183].

A 2015 meta-analysis investigating 19 observational studies conducted in children with obesity reported altered plasma LC-PUFAs, particularly concerning ω-6 series, as well as higher LA conversion rate, reduced Δ5 desaturase activity and increased AA levels, which are correlated with metabolic dysregulation [184].

In recent years, increasing attention has been paid to dietary fatty acids’ role in the gut microbiota. Dietary pattern can influence the composition of the gut microbiota, and recent research has suggested that it impacts health and contributes to chronic degenerative disease pathogenesis [185,186,187,188]. “Western-style” dietary patterns high in saturated fats and simple sugars and poor in fiber and bioactive molecules impair the gut microbiota composition and affect its metabolism, including reduced production of intestinal short-chain fatty acids (SCFAs), particularly butyrate, thus contributing to the development of IR [185,186].

Preliminary data from a recent study on the effect of 4 months of 500 mg/day DHA supplementation, combined with dietary intervention and lifestyle education, on the gut microbiota and metabolic biochemical parameters in a pediatric population showed a decrease in the Firmicutes/Bacteroidetes ratio (F/R ratio) that was even more evident post-supplementation [189,190,191,192]. The results of this study suggest a strong impact of DHA on the gut microbiota regardless of dietary and lifestyle intervention, although further data are needed to derive definitive conclusions.

**Figure 4:**
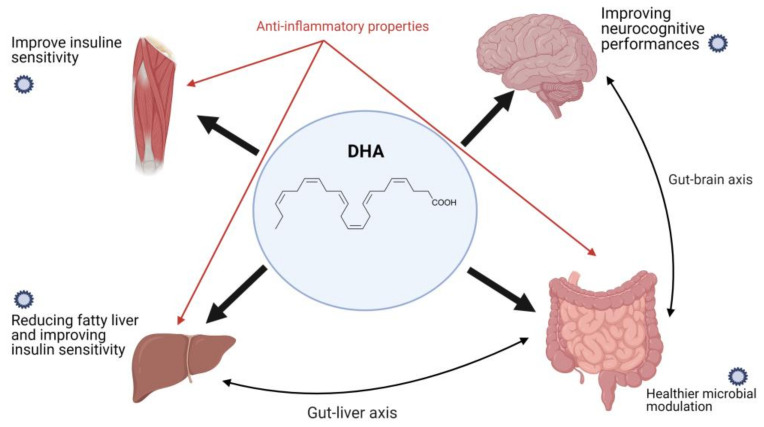
Anti-inflammatory properties of DHA and its effects on brain, liver, gut and skeletal muscle. Created with BioRender.com (accessed on 26 May 2021). DHA: Docosahexaenoic acid.

The anti-inflammatory properties of DHA and its effects on various organs are shown in Figure 4.

### 7.3. Red Yeast Rice (RYR)

Fermented red yeast rice (RYR), obtained from the fermentation of rice (*Oryza sativa*) by the yeast *Munascus purpureus*, is an ancient supplement from the East. It is rich in bioactive compounds (monacolines) with a lowering effect on plasma lipids and LDL-C [193,194].

It also appeared to have significant effects on endothelial function, measured as pulse volume (PV), and arterial stiffness, measured as pulse wave velocity (PWV), in 40 moderately hypercholesterolemic subjects [195]. In a prospective study of 5000 adult patients with myocardial infarction, supplementation with RYR also appeared to reduce the incidence of coronary events [194].

RYR is often combined with other supplements or botanicals (berberine or polycosanols) exerting positive effects on cholesterol, triglyceride and glucose plasma levels as well as adiponectin/leptin ratio [196]. The limitation of this evidence is that these studies were all carried out on adults, so further studies on younger populations are needed to clarify the potential effect of this nutraceutical on young patients suffering from PCOS.

### 7.4. Berberine (BBR)

A recent review investigated the therapeutic effect of berberine (BBR) on PCOS [197]. Indeed, this substance, which is an isoquinoline alkaloid, is a main component of many commonly used botanicals, such as *Coptis chinensis Franch* [198]. Berberine has shown many functions, including lowering blood glucose, blood lipid regulation, coronary arteries dilatation, antibacterial activity and antiarrhythmic properties [197]. Fifteen potential targets of BBR have been identified, including aldo-keto reductase family 1 member C3 (AKR1C3), insulin receptor, estrogen receptor and tyrosine-protein phosphatase non-receptor type 1. This substance appears to be able to activate the insulin signaling pathway by means of some key proteins. It promotes glucose transport and consumption and lipid metabolism, impacting lipid synthesis; increases anti-inflammatory cytokine secretion; alleviates low-grade chronic inflammation; suppresses oxidative stress; and shapes the gut microbiota, significantly reducing the Firmicutes and Bacteroides proportion [197]. Therefore, BBR has been suggested as an adjuvant in the medical treatment of PCOS; however, further studies addressing combinations of different molecules with antioxidant and/or anti-inflammatory properties in adolescents with PCOS are needed.

### 7.5. Curcumin

Curcumin is known to have several anti-inflammatory properties; by acting on the capacity of superoxide dismutase, it can regulate cytokines, protein kinases and certain adhesion molecules. Curcumin has been shown to be effective in metabolic syndrome [199]. It acts as an insulin sensitizer in animal models [200], while in RCT studies on subjects supplemented with 1500 mg for 3 to 9 months, it was able to reduce the rate of T2D onset, besides impacting cytokine secretion, adiponectin increase and leptin decrease [201,202] and lowering plasma lipids through increased expression of ATP-binding cassette transporter A1 (ABCA1) and apolipoprotein (APO)A-I [203]. 

Moreover, when women with PCOS were supplemented with curcumin in combination with metformin, either 80 or 500 mg per day three times a day for 3 months, the authors reported a significant reduction in HOMA-IR, QUICKI and LDL-C and an HDL-C increase [204]. 

Although curcumin is well tolerated in humans, its poor bioavailability is a major limitation in clinical practice. For this reason, several formulations have been developed [205], including milk exosomes encapsulation in order to improve gastric resistance and increase intestinal absorption as well as combination with piperine, which promotes hepatic glucuronidation [206].

## 8. Prebiotics, Probiotics and Postbiotics

In recent years, the use of probiotics, prebiotics or synbiotics (products containing pre- and probiotics together) to improve interactions between the gut microbiota and host metabolism in different chronic degenerative diseases has been investigated. A recently published review examined the effects of probiotics and synbiotics on obesity, IR, T2D and non-alcoholic fatty liver disease (NAFLD) [200].

Probiotics are live microorganisms that may have some beneficial effects on gut microbiota composition and intestinal balance. Specific bacterial species such as Bifidobacterium spp. have been shown to improve glucose homeostasis, reduce weight gain and fat mass and restore insulin secretion. Prebiotics, on the other hand, are substances that beneficially affect the host by selectively stimulating the growth and/or activity of bacteria that colonize the large intestine. These are mainly short-chain oligosaccharides or polysaccharides, contained in common plant-based foods such as vegetables, unrefined cereals and fortified foods. The most well-known prebiotics are fructosyl-oligosaccharide (FOS), galactosyl-oligosaccharide (GOS) and other oligosaccharides found in milk, which are converted by the intestinal microbiota into SCFAs and simultaneously promote specific commensal bacterial proliferation in the colon. For example, inulin is known to stimulate the growth of bifidobacteria and can reduce energy intake and body fat mass [207]. Stimulation of bifidobacteria growth by prebiotics correlates with an increase in glucose tolerance and glucose-mediated insulin secretion and normalizes inflammation. In particular, supplementation with GOS decreases hepatic triglyceride levels by reducing the activity of enzymes involved in the synthesis of fatty acids and proteins involved in the synthesis of very-low-density lipoprotein (VLDL) [208].

In overweight adults, studies on probiotic intervention have revealed a positive effect on glucose metabolism, BMI and fat mass. Indeed, a 6-week randomized controlled trial of 60 overweight Indian individuals found that the probiotic mixture VSL3 improved glucose balance [209]. Moreover, a double-blind RCT on 44 obese children with 4-month supplementation of VSL#3 showed significantly improved NAFLD [210].

In another RCT in obese adolescents, supplementation with L. salivarius Ls-33 led to a significant increase in the Bacteroidetes/Firmicutes ratio but had no effect on MetS [211]. Miccheli et al. demonstrated that the administration in obese children and adolescents of a probiotic composed of a large concentration of eight free-dried live bacterial species, all normal components of the human gastrointestinal microbiota, including four strains of lactobacilli (L. casei, L. plantarum, L. acidophilus and L. delbrueckii subsp. bulgaricus), three strains of bifidobacteria (B. longum, B. breve and B. infantis) and S. salivarius subsp. Thermophilus induced changes in the host’s urinary and intestinal metabolism of certain amino acids, nucleic acid degradation and creatinine metabolism, reducing the risk of NAFLD [212].

In a triple-blind randomized trial with 64 obese children who received a probiotic capsule (with Lactobacillus acidophilus, Bifidobacterium lactis, Bifidobacterium bifidum and Lactobacillus rhamnosus) for 12 weeks, an improvement in the grading of NAFLD and a reduction in AST and ALT, total cholesterol, LDL and triglycerides were observed [213]. In another RCT, 22 obese children (8–12 years) with supplementation of *Lactobacillus* GG for 8 weeks showed a decrease in ammino transferase and changes in BMI z-score and visceral fat [214].

Evidence of the anti-obesity or MetS treatment effect of prebiotics and probiotics is still lacking, especially in children. In particular, accurately designed studies using a specific and appropriate dose of probiotics or prebiotics and a controlled diet would be valuable in assessing individual responses to different types of intervention as well as the influence of genetic, environmental and microbial factors [215].

Postbiotics are substances released by or produced through the metabolic activity of the microorganisms that directly exert beneficial effects on the host [216].

Postbiotics are designed to mimic the effect of probiotics by avoiding the risk of administering live microorganisms. They comprise a variety of molecules, including lipids, proteins, carbohydrates, SCFAs, peptides, enzymes, teichoic acids, vitamins and co-factors, organic acids and molecular complexes such as peptidoglycan and lipoteichoic acids [217]. The main strains used as postbiotics are Lactobacillus and Bifidobacterium, but emerging studies also involve Streptococcus and Faecalibacterium species [218,219].

The most important SCFAs are acetate, propionate and butyrate [217]. Butyrate and propionate have been shown to stimulate intestinal hormones and reduce food intake [218]. In addition, propionate appears to inhibit lipogenesis by downregulating hepatic fatty acid synthesis, whereas acetate acts as a lipogenic substrate; thus, the acetate/propionate ratio is critical for de novo lipogenesis [219]. Propionate and butyrate can also induce the expression of gluconeogenesis-related genes by activating intestinal gluconeogenesis through various mechanisms, resulting in reduced body weight and adiposity [220]. Another promising postbiotic is KetoA, a metabolite produced from linoleic acid by lactic acid bacteria in the gut, with the potential to increase energy expenditure [221]. In view of the increasingly central role of the gut microbiota in the pathogenesis of the low-grade inflammation that determines obesity and how gut dysbiosis conditions the onset of MetS in obese subjects, it would be desirable to conduct clinical trials in the pediatric population affected by obesity and MetS to assess the effects of supplementation with postbiotics, combined with interventions aimed at improving diet and lifestyle, on the composition of the gut microbiota and anthropometric and metabolic parameters. A recent systematic review examining the role of prebiotics, probiotics, postbiotics and synbiotics in the management of adult obesity found poor results for MetS, with little change in BMI or body weight. Limitations are also reported, such as the poor heterogeneity between study subgroups of research conducted in children and the need for further large-scale RCTs in humans to assess the beneficial properties of probiotics, prebiotics, synbiotics and postbiotics in order to establish the ideal dose; the duration of supplementation and the duration of their beneficial effects; and their safety profile in the prevention and management of obesity [222].

## 9. Emerging Functional Foods and Food Supplements

Recently, some functional foods have attracted significant interest for the treatment of IR and for the control of carbohydrate metabolism.

For example, the well-known medicinal plant *Lagerstroemia speciosa* (commonly called *banaba*) has been studied extensively over the last century for its properties on glucidic and lipid regulation and has been shown to have hypoglycemic and hypolipidemic effects on insulin and HbA1c in mice with T2D. The effect appears to be dependent on some compounds present in the plant: corosolic acid and ellagitannine [223]. Some studies conducted in mice showed that supplementation with corosolic acid (from 2 to 10 mg/kg) led to a reduction in glucose levels and increased the expression of GLUT4 [224,225].

Furthermore, the well-known sweet potato, *Ipomea batatas* (L.), contains several phytochemical compounds such as triterpenes, alkaloids, coumarins, flavonoids, saponins, tannins and phenolic acids [226]. Due to its properties, it is used by different populations for the treatment of T2D. In addition, when administered in powder form, it appears to have positive effects on insulin levels in diabetic mice, and if taken for at least 6 weeks, circulating levels of triglycerides (TG) and free fatty acid (FFA) are reduced [227,228,229].

Finally, flaxseeds, already widely known for their properties since they are rich in alpha-linoleic acid (ALA), have received greater interest in the presence of a class of phytoestrogens with positive effects on human health—lignans. Being phytoestrogens, they produce estrogen-like effects in mammals. It has been shown that lignans have numerous biological activities [230,231,232]. They reduce glucose concentration, blood pressure and plasma cholesterol. When supplemented in obese mice fed a high-fat diet (HFD), they showed a reduction in visceral fat, a decrease in insulin and a lower concentration of total cholesterol [233,234].

Although this early evidence is based on animal models, it appears to be promising. Clinical trials are therefore needed to clarify the efficacy of these compounds in the management of IR in PCOS patients in order to expand therapeutic possibilities.

**Figure 5:**
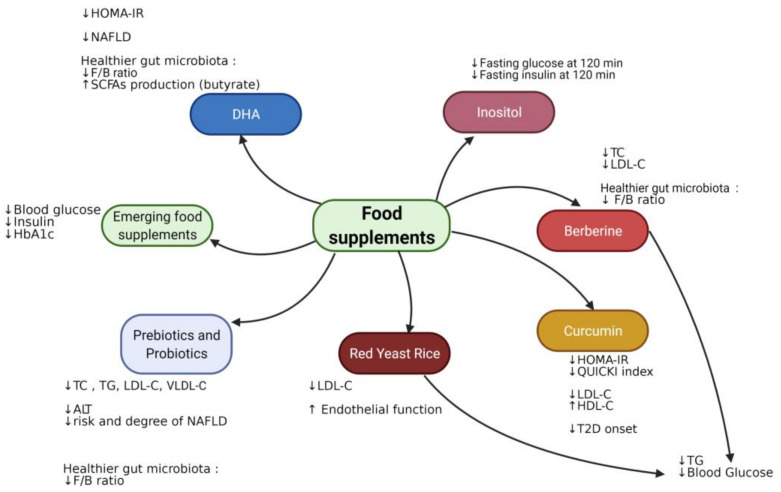
Food supplements and their effects on glucidic and lipidic metabolism and gut microbiota. Created with BioRender.com (accessed on 26 May 2021). HOMA-IR: homeostatic model assessment for insulin resistance; DHA: Docosahexaenoic acid; NAFLD: non-alcoholic fatty liver disease; SCFAs: short-chain fatty acids; TC: total cholesterol; TG: tryglicerides; LDL-C: low-density lipoprotein cholesterol; VLDL-C: very low-density lipoprotein cholesterol; ALT: alanine aminotransferase; HDL-C: high-density lipoprotein cholesterol; QUICKI index: quantitative insulin-sensitivity check index; F/B ratio: Firmicutes (F) and Bacteroidetes (B); T2D = type 2 diabetes; ↑increased; ↓decreased.

In Figure 5, the effects of nutraceuticals on glucidic and lipidic metabolism and gut microbiota are reported.

## 10. Conclusions

PCOS is associated with important reproductive morbidity. The association of IR with this endocrine reproductive pathology has been well documented. Early diagnosis and precocious treatment are crucial to limit ovarian failure. Making use of the benefits of nutrition therapy and food supplementation on IR and other risk factors may be considered as a preventive strategy to restore ovulation and to protect fertility in adolescents.

## Figures and Tables

**Table 1:** Diagnostic Criteria for PCOS in Adolescents [18,19,32].

Required
Ovulatory dysfunction: Abnormal menstrual pattern for age/gynecologic age, persistent for 1–2 years, as: - Amenorrhea or - Oligomenorrhea or - Excessive uterine bleeding and Hyperandrogenism: - Biochemical: elevation of total/free serum testosterone *or* - Clinical: moderate–severe hirsutism
**Not Recommended**
PCOM Obesity IR/hyperinsulinism Severe cystic acne Biomarkers (AMH, T/DHT)

PCOM = polycystic ovarian morphology; AMH = anti-Müllerian hormone; T = testosterone; DHT = dihydrotestosterone.

## Data Availability

Not applicable.
